# Novel Sonic Hedgehog Mutation in a Couple with Variable Expression of Holoprosencephaly

**DOI:** 10.1155/2011/703497

**Published:** 2011-09-08

**Authors:** M. Aguinaga, I. Llano, J. C. Zenteno, S. Kofman Alfaro

**Affiliations:** ^1^Genetics Department, Instituto Nacional de Perinatología, Montes Urales 800. Col. Lomas Virreyes, 11000 México, DF, Mexico; ^2^Genetics Department and Research Unit, Instituto de Oftalmología “Conde de Valenciana”, Avenida Chimalpopoca 14, Colonia Obrera, 06800 México, DF, Mexico; ^3^Genetics Department, Hospital General de México, Dr. Balmis 148, 06726 Mexico, DF, Mexico

## Abstract

Holoprosencephaly (HPE) is the most common developmental defect of the forebrain and midface in humans. sporadic and inherited mutations in the human sonic hedgehog (SHH) gene cause 37% of familial HPE. A couple was referred to our unit with a family history of two spontaneous first trimester miscarriages and a daughter with HPE who presented early neonatal death. The father had a repaired median cleft lip, absence of central incisors, facial medial hypoplasia, and cleft palate. Intelligence and a brain CT scan were normal. Direct paternal sequencing analysis showed a novel nonsense mutation (W127X). Facial characteristics are considered as HPE microforms, and the pedigree suggested autosomal dominant inheritance with a variable expression of the phenotype. This study reinforces the importance of an exhaustive evaluation of couples with a history of miscarriages and neonatal deaths with structural defects.

## 1. Introduction

Holoprosencephaly (HPE) is the most common developmental defect of the forebrain and midface in humans [1], and it is a frequent cause of prenatal death, with an estimated frequency of 1/250 abortions and 1/16,000 live births [2]. In about 70–80%, the severity of the brain malformations is accompanied by characteristic craniofacial abnormalities [3]. Clinical expression is variable, alobar HPE with cyclopia represents the most severe end of the spectrum, and various microforms have been described which include cleft lip and palate, single maxillary incisor, ocular hypotelorism, iris coloboma, flat nose, absent frenulum and midface hypoplasia [4–6]. 

Several genes are implicated in the pathogenesis of HPE. Sporadic and inherited mutations in the human Sonic Hedgehog (*SHH*) gene have been shown to cause holoprosencephaly (HPE3 (MIM 236100)) in 3.7% of sporadic, 18% of familial cases and 37% families with autosomal dominant transmission [7]. 

Mutations and microdeletions in other genes (*SIX3*, *ZIC2*, and* TG1F*), and environmental factors such as maternal diabetes, alcohol, and retinoic acid exposure during pregnancy as well as some monogenic syndromes can also cause HPE [8, 9]. 

Here, we describe a novel *SHH* mutation in the male partner of a couple with pregnancy loss and a daughter with a severe expression in the phenotype.

## 2. Case Presentation

**Figure 1:**
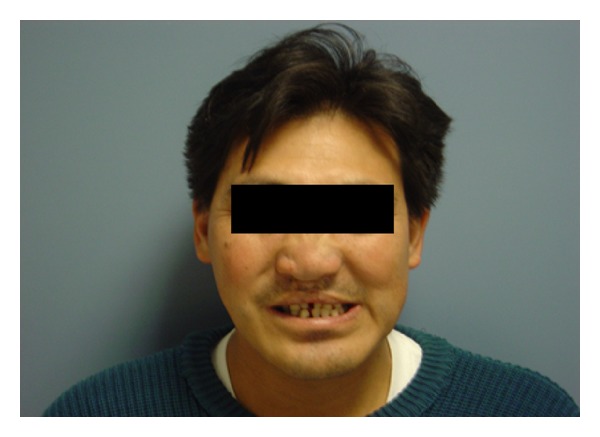
Facial patient's profile. Note absence of central incisor, repaired cleft lip and midface hypoplasia.

A couple was referred to the Genetics Reproductive Clinic with a family history of two spontaneous first trimester miscarriages and a daughter with holoprosencephaly and cebocephaly who presented early neonatal death. Parents were nonconsanguineous, and the father had a repaired median cleft lip, absence of central incisors, facial medial hypoplasia, and cleft palate (Figure 1). Intelligence and a brain CT scan were normal. The mother was a healthy 27-year-old woman. Karyotype was normal.

After obtaining local ethics institutional approval and informed consent, blood samples were drawn from the couple by venipuncture, and genomic DNA was extracted using the GFX Genomic Purification kit (GE HealthCare, Biosciences). The complete *SHH* coding sequence including the exon intron boundaries was amplified by PCR (primer sequences available upon request). Direct automated sequencing was performed with the Big Dye Terminator Cycle Sequencing kit (Applied Biosystems, Foster City, Calif, USA) in an ABI Prism 310 Genetic Analyzer (Applied Biosystems).

**Figure 2:**
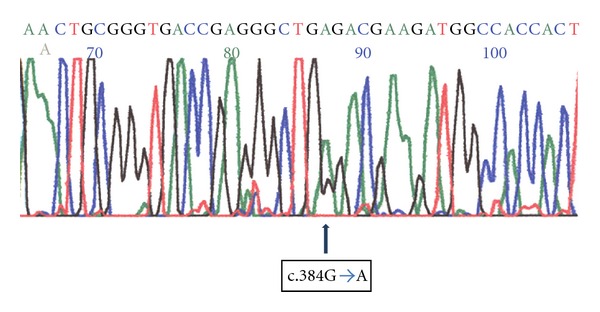
Nonsense heterozygous mutation at codon 127 of the *SHH* gene (W127X). This mutation causes a G for A transition at cDNA nucleotide 384 causing a substitution of tryptophan (TGG) for a stop codon (TGA) in exon 2 (c.384G→A).

Direct sequencing analysis of the complete *SHH* ORF in DNA from the father showed a nonsense mutation at codon 127 (W127X) (Figure 2). This mutation consists of a G for A transition at cDNA nucleotide 384 causing a substitution of tryptophan (TGG) for a stop codon (TGA) in exon 2 (c.384 G→A). The mother's *SHH* ORF had a normal sequence. We were not able to perform molecular analysis in the miscarriages or previous child of the couple.

## 3. Discussion

We report a novel nonsense mutation in a father with a family history of two miscarriages and a previous child with a severe HPE phenotype. The patient presented minor signs localized to the facial region characterized by a median cleft lip, absence of central incisor, medial facial hypoplasia, and cleft palate. These facial characteristics are considered microforms of HPE, and the pedigree suggested autosomal dominant inheritance. Other families with this type of transmission and wide phenotypic variability have been described [5, 7, 10]. 

Nanni et al. [6] studied 78 families with HPE in which nine of them had mutations in the *SHH* gene. They observed that in four families, the *SHH* mutation was present in a nonaffected parent. 

Marini et al. [10] reported a family with recurrence of autosomal dominant HPE in different members showing a wide clinical variability. They identified a nonsense mutation at codon 128 (W128X) with a change of TGG for TAG in the mother and three severely affected children. The mutation found in this case is at position 127 (TGG for TGA), which suggests that mutations in this adjacent residues are associated with an important phenotypic clinical variability. 

The etiology of the wide phenotypic spectrum is not yet understood, and it has been proposed that other genes or environmental influences may contribute as gene modifiers to the phenotype [11, 12]. Nanni et al. [6] found three patients with mutations in the *SHH* gene and in other genes such as *TGIF* and *ZIC2*. In the present study, mutation analysis in the HPE child was not possible, so we cannot exclude the possibility of a digenic inheritance. 

Previous studies have suggested a modifier gene in the X chromosome based on a predominant female transmission and the observation that most affected patients were male [6, 10, 13, 14]. Odent et al. [15] found a significant difference (*P* = 0.002) in the gender of the transmitting parent, observing that in 14 of 16 families studied, the mother had the mutation. They suggested that males with HPE may have a diminished reproductive fitness which can be interfering with their reproductive life. The present case shows the vertical transmission of a father to his daughter, not supporting previous observations. 

This study reinforces the importance of evaluating patients with miscarriages and neonatal deaths with structural defects, searching for microforms in couples with a previous child with HPE. The molecular analysis allowed us to give a recurrence risk to the couple, which due to the variability in expression, has to include all the possible clinical forms of HPE.

## References

[1] Matsunaga E, Shiota K (1977). Holoprosencephaly in human embryos: epidemiologic studies of 150 cases. *Teratology*.

[2] Cohen MM (1989). Perspectives on holoprosencephaly: part I. Epidemiology, genetics, and syndromology. *Teratology*.

[3] Corsello G, Buttitta P, Cammarata M (1990). Holoprosencephaly: examples of clinical variability and etiologic heterogeneity. *American Journal of Medical Genetics*.

[4] Belloni E, Muenke M, Roessler E (1996). Identification of Sonic hedgehog as a candidate gene responsible for holoprosencephaly. *Nature Genetics*.

[5] Roessler E, Ward DE, Gaudenz K (1997). Cytogenetic rearrangements involving the loss of the Sonic Hedgehog gene at 7q36 cause holoprosencephaly. *Human Genetics*.

[6] Nanni L, Ming JE, Bocian M (1999). The mutational spectrum of the Sonic Hedgehog gene in holoprosencephaly: SHH mutations cause a significant proportion of autosomal dominant holoprosencephaly. *Human Molecular Genetics*.

[7] Wallis D, Muenke M (2000). Mutations in Holoprosencephaly. *Human Mutation*.

[8] Cohen MM, Shiota K (2002). Teratogenesis of holoprosencephaly. *American Journal of Medical Genetics*.

[9] Bendavid C, Dubourg C, Gicquel I (2006). Molecular evaluation of foetuses with holoprosencephaly shows high incidence of microdeletions in the HPE genes. *Human Genetics*.

[10] Marini M, Cusano R, De Biasio P (2003). Previously undescribed nonsense mutation in SHH caused autosomal dominant holoprosencephaly with wide intrafamilial variability. *American Journal of Medical Genetics*.

[11] Ming JE, Muenke M (2002). Multiple hits during early embryonic development: digenic diseases and holoprosencephaly. *American Journal of Human Genetics*.

[12] Suthers G, Smith S, Springbett S (1999). Skewed sex ratios in familial holoprosencephaly and in people with isolated single maxillary central incisor. *Journal of Medical Genetics*.

[13] Pineda-Alvarez DE, Dubourg C, David V, Roessler E, Muenke M (2010). Current recommendations for the molecular evaluation of newly diagnosed holoprosencephaly patients. *American Journal of Medical Genetics, Part C*.

[14] Roessler E, Belloni E, Gaudenz K (1996). Mutations in the human Sonic Hedgehog gene cause holoprosencephaly. *Nature Genetics*.

[15] Odent S, Attié-Bitach T, Blayau M (1999). Expression of the Sonic hedgehog (SHH) gene during early human development and phenotypic expression of new mutations causing holoprosencephaly. *Human Molecular Genetics*.

